# Prebiotic Peptides Based on the Glycocodon Theory Analyzed with FRET

**DOI:** 10.3390/life11050380

**Published:** 2021-04-23

**Authors:** Jozef Nahalka, Eva Hrabarova

**Affiliations:** 1 Institute of Chemistry, Centre for Glycomics, Slovak Academy of Sciences, Dubravska Cesta 9, SK-84538 Bratislava, Slovakia; chemhra@savba.sk; 2 Institute of Chemistry, Centre of Excellence for White-Green Biotechnology, Slovak Academy of Sciences, Trieda Andreja Hlinku 2, SK-94976 Nitra, Slovakia

**Keywords:** prebiotic chemistry, protein–monosaccharide recognition, protein–monosaccharide interactions, FRET analysis, glycocodon theory, glucose oxidase

## Abstract

In modern protein–carbohydrate interactions, carbohydrate–aromatic contact with CH–π interactions are used. Currently, they are considered driving forces of this complexation. In these contacts, tryptophan, tyrosine, and histidine are preferred. In this study, we focus on primary prebiotic chemistry when only glycine, alanine, aspartic acid, and valine are available in polypeptides. In this situation, when the aromatic acids are not available, hydrogen-bonding aspartic acid must be used for monosaccharide complexation. It is shown here that (DAA)n polypeptides play important roles in primary “protein”–glucose recognition, that (DGG)n plays an important role in “protein”–ribose recognition, and that (DGA)n plays an important role in “protein”–galactose recognition. Glucose oxidase from *Aspergillus niger*, which still has some ancient prebiotic sequences, is chosen here as an example for discussion.

## 1. Introduction

The origin and evolution of modern biochemistry and cellular life are a very interesting field of research; each human generation postulates new theories. Unfortunately, a top-down approach from the perspectives of modern biochemistry and molecular biology is easier to formulate than a bottom-up approach starting from the physicochemical properties of nucleic and amino acid polymers [1]. Nevertheless, 50 years of research exploring the origin and early evolution of life has created the concepts of an “RNA world” [2,3], a “protein world” [4,5], and a “protein–RNA coevolution world” [6,7] and many other hypotheses; however, the role of sugars is still relatively underestimated. After nucleotides and amino acids, sugars can be considered the third alphabet of life and are used to transfer information from the cellular environment via protein–glycan interactions [8]. Cells are covered by a “sugar coat” (glycocalyx) attached to membrane proteins and lipids; the carbohydrate moieties of membrane glycolipids and glycoproteins provide a first line of contact to the surrounding environment and are unique for different bacterial colonies or animal cells/tissues. The gel-like sugar layer has protective and sensing functions; for example, the glycocalyx of vascular endothelial cells serves as a negatively charged molecular sieve and acts as a signaling platform [9,10]. Eukaryotes, and some bacteria, adopt the “sugar code” to protein synthesis; N-linked protein glycosylation is performed in the endoplasmic reticulum (ER); and the N-linked glycosyl moiety controls protein folding and finalization. Different cells/tissues provide different N-glycosylation patterns/signatures; therefore, the protein expression is highly specific and controlled; when out of control, polypeptides aggregate and form inclusion bodies, which is the main problem in recombinant protein production. Oligosaccharides are assembled at the ER membrane on a lipid carrier by glycosyltransferases, and then transferred to asparagine (N-X-S/T sequons) of the polypeptide chain by an oligosaccharyltransferase [8,11]. The activity and composition of glycosyltransferases are specific for each cell/tissue.

In early prebiotic chemistry, when the first peptide–monosaccharide interaction was selected and conserved, the number of different amino acids in short peptides and the number of three-dimensional structures were limited; in light of this, minimal amounts of amino acids and minimally ordered peptide chains were used for primary interactions and for primary glyco-code evolution. It seems that, today, N-linked protein glycosylation in the ER uses evolved glyco-codes to control protein folding and finalization. On the other hand, our previous work gave a number of examples from “modern” proteins in which disordered protein chains recognize/bind the monosaccharide. For example, in the Bacteriocin LLPA–mannose complex (3M7J), a disordered QGDGN175 sequence recognizes and binds mannose [12,13]. The glycocodon theory states that the monosaccharide units of a 3D glycan structure are recognized by the glycocodons, which are short amino acid sequences (sequons) composed of a polar/aromatic amino acid and an amino acid couple specific for the monosaccharide [12,13]. 

**Figure 1:**
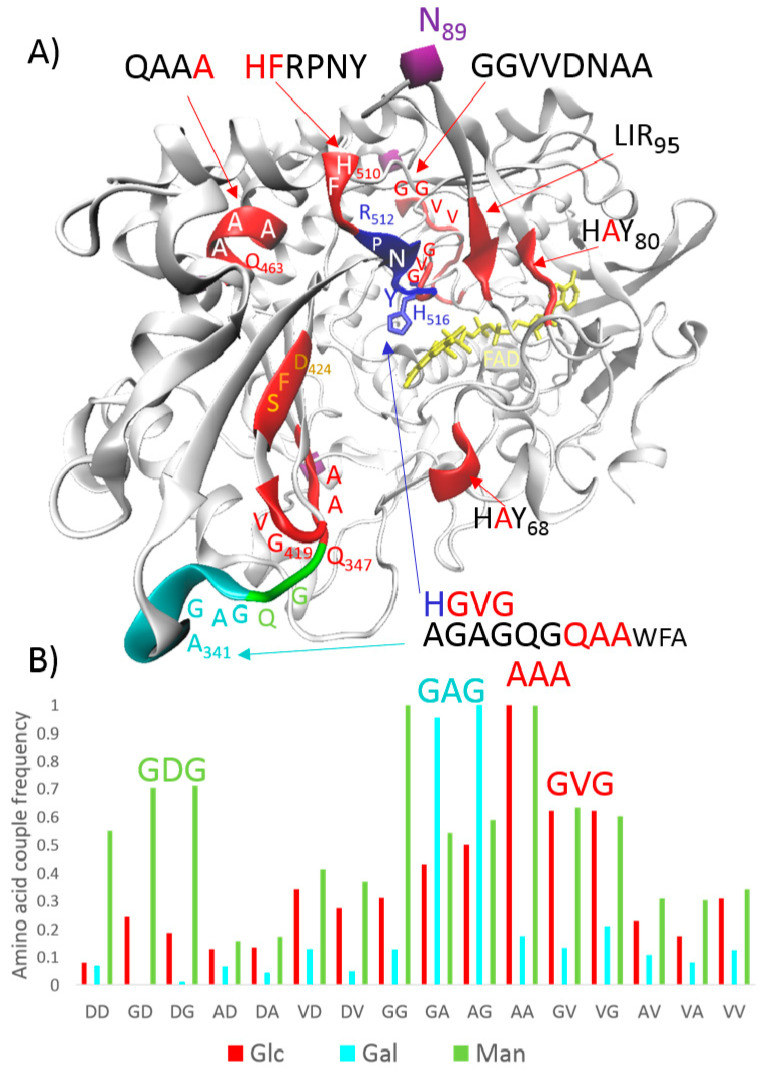
An explanation of the glycocodon theory [12]. (**A**) GO monomer—glucose oxidase from *Aspergillus niger*. N89—glycosylation site for an extended carbohydrate moiety forming a bridge between two monomers. GO is inactive without this N-linked glycosyl moiety. Red highlights—sequons/glycocodons for glucose (Glc); cyan highlights—ancient sequons/glycocodons for galactose (Gal); green highlights—ancient sequons/glycocodons for mannose (Man); and blue highlights—catalytic area with a key histidine. Protons are transferred from C1-Glc to H516 [15]. (**B**) Frequencies of amino acid couples, composed of G, A, D, and V amino acids, in monosaccharide-specific proteins, data from [12].

Ikehara proposed a prebiotic protein world enriched with peptides/proteins composed of glycine, alanine, aspartic acid, and valine (G, A, D, and V, respectively; also abbreviated as Gly, Ala, Asp, and Val) [4,5]. A simple BLAST search against the human genome for decapeptides with a combination of runs composed of two amino acids showed that the G, A, D, and V amino acids are extremely overrepresented [14]. In light of this, the first glycocodons were derived from ancient prebiotic conditions when only the G, A, D, and V amino acids were available in building primary proteins. For example, DAA (Asp-Ala-Ala) and DGV (Asp-Gly-Val) were proposed for glucose (Glc), DGG (Asp-Gly-Gly) was proposed for ribose (Rib), (G)DGD (Asp-Gly-Asp) was proposed for mannose (Man), and DAG (Asp-Ala-Gly) was proposed for galactose (Gal) [12,13]. These proposals were based on the measured frequencies of G, A, D, and V amino acid couples in monosaccharide-specific proteins (e.g., 6321 proteins specific for Glc) and were verified on the protein–monosaccharide structures available in the Protein Data Bank (PDB) [12]. Figure 1 depicts the measured frequencies for Glc, Gal, and Man. Glucose oxidase (GO) from *Aspergillus niger* is shown in the figure as an example of conserved ancient sequences.

The GO monomer has a deeply buried flavin adenine dinucleotide cofactor (FAD/FADH_2_, ≈15 Å from the surface), and the open, deep pocket with a dimension of ≈10 Å × 10 Å provides access to the active site [15]. Access is covered by the glycocodons, which attract Glc towards the catalytic area (Figure 1A). In the catalytic area, Glc molecules form hydrogen bonds with R512, N514, H516, and Y68; the O1 hydroxyl group of Glc is located close to the key H516; and protons are transferred from C1-Glc to H516 [15]. In GO, H516GV(G) is an example glycocodon, which is a short, disordered chain, and helps organize the catalytic area in preparation for a reaction (Figure 1A).

In this study, we constructed FRET fusion proteins composed of a cyan fluorescent protein (CFP) and a yellow fluorescent protein (YFP), a common pair of fluorophores for the biological use of FRET (fluorescence resonance energy transfer). The mentioned color variants of the green fluorescent protein were fused using the peptide linker composed of a triple tandem of glycocodons for Rib, Glc, Gal, and Man. The results are discussed in the next sections; it is shown that the FRET signal changes in accordance with the glycocodon theory.

## 2. Materials and Methods

### 2.1. Cloning and Expression

**Figure 2:**
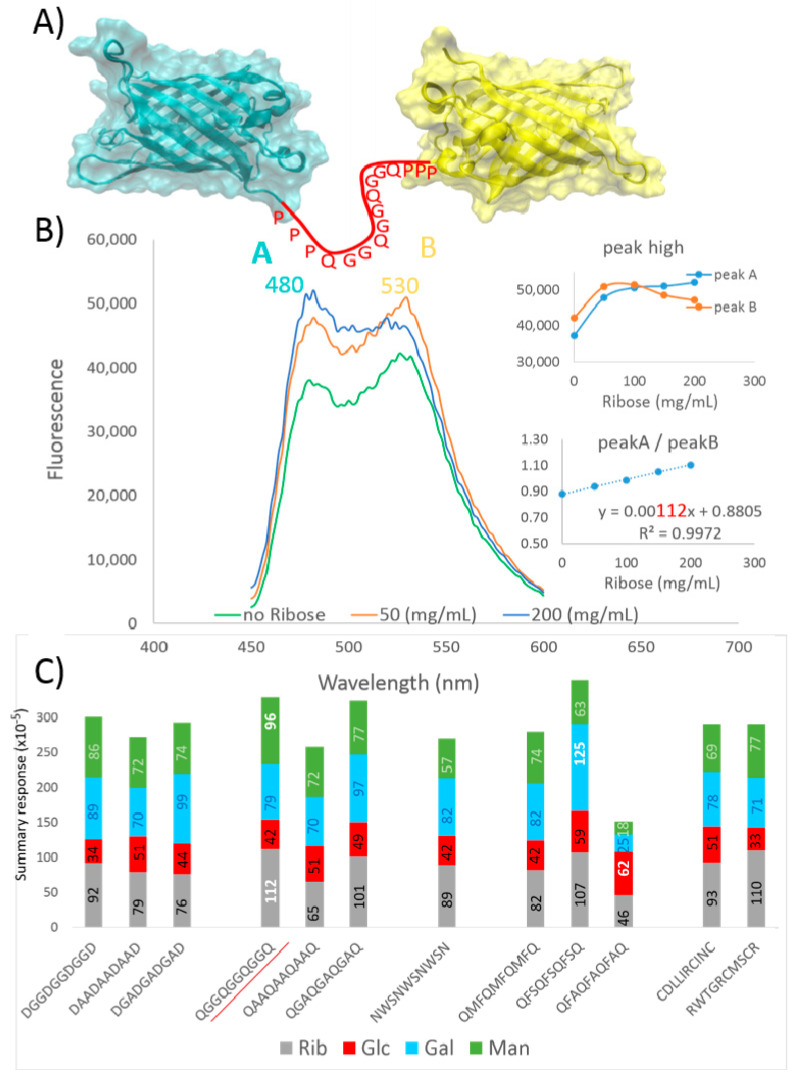
Peptides based on the glycocodon theory analyzed with FRET. (**A**) Illustration of the protein constructs used: cyan fluorescent proteins (CFP) and yellow fluorescent proteins (YFP) are linked by PPP-(glycocodon)_3_-PPP linkers. (**B**) In the FRET constructs, the CFP-donor emits light at the 480 nm wavelength and the YFP-acceptor emits light at the 530 nm wavelength. The slope of change in the ratio between peak high A vs. peak high B, which are dependent on monosaccharide concentration, was used for the evaluation. (**C**) Responses of various linkers to increasing concentrations of the monosaccharides: red—glucose (Glc), cyan—galactose (Gal), green—mannose (Man), and grey—ribose (Rib). The summary response shows how the linker reacts to the monosaccharides tested overall; the monosaccharide-specific FRET responses (×10^–5^) are color-coded within the columns.

For CFP-YFP-fused constructs, the mTurquoise2 and SYFP2 protein sequences were used (https://www.fpbase.org (accessed on 22 April 2021)). Codon optimization for *E. coli*, gene synthesis and cloning to the pET-51b (+) plasmid were performed using GenScript. Ten amino acids in the fusion linker were then mutated to the tested linkers (Figure 2C) using GenScript. Chemically competent *E. coli* BL21 (DE3)T1R cells were transformed with the plasmids and cultivated on ampicillin/LB/agar plates. The colonies were regrown in 30 mL LB medium supplemented with ampicillin (150 g/L) for approximately 20 h at 36 °C; then, 15 mL of the solution was transferred to 100 mL for 4 h at 37 °C and 150 rpm; and the recombinant expression was accomplished by adding an inductor (IPTG; 95.2 g/L) for 20 h at 20 °C and 150 rpm. After centrifugation (30 min, 4000× *g* rpm, and +4 °C), the biomass was suspended in a minimal volume of water and immediately lyophilized. The yields of the dry cells varied from 120 to 150 mg per cultivation flask. The lyophilized cells were kept at −30 °C until further use.

### 2.2. Isolation of FRET Constructs

The FRET mutant variants—each of the two parallels (25 mg)—were lysed in Bug Buster Protein Extraction Reagent (Novagen, Darmstadt, Germany) detergent, cooled on ice, and centrifuged, and the cleared lysates were loaded on a 1 mL Strep-Tactin Superflow Plus Cartridge (QIAGEN, Hilden, Germany). According to the QIAGEN protocol, the proteins were washed and isolated in a volume of 4 mL of Strep-Tactin elution buffer (pH 8.0). The concentration of proteins was assayed using a Total Protein Kit, Micro Lowry, Onishi & Barr Modification (Sigma-Aldrich, St. Louis, MO, USA). The elution products were kept at −30 °C until further use.

### 2.3. The Protein–Monosaccharide Interactions and FRET Analysis

The protein–monosaccharide interactions were investigated using the measurement of fluorescence spectra using a TECAN Infinite M200 apparatus. Prior to this, all of the mutants were diluted with a Strep-Tactin washing buffer (pH 8.0) to achieve as comparable a starting fluorescence as possible. The sugars were weighed (5, 10, 15, and 20 mg) and dissolved in 100 µL diluted protein solutions. Afterwards, they were kept at +4 °C overnight to achieve an optimal steady state, and after centrifugation (1 min, 13,000× *g* rpm, and +4 °C), 100 µL were pipetted onto a 96-well black microplate. Additionally, reference samples of all of the protein mutants were measured without sugar.

## 3. Results

The results are depicted in Figure 2. As can be seen, the studied peptide is connected to the C-end of CFP and to the N-terminus of YFP by three prolines (Figure 2A). The CFP-(QGG)_3_Q-YFP construct and the influence of Rib on FRET is shown as an example (Figure 2B). The CFP-donor emits light at 480 nm wavelength, and the YFP-acceptor emits light at 530 nm wavelength. In the FRET concept, the FRET efficiency depends mostly on the distance between the CFP-donor and the YFP-acceptor; therefore, the acceptor emission is maximal at the shortest distance between the CFP and YFP domains. When the monosaccharide is in the contact with the linker and is inserted between the domains, the acceptor emission decreases and the donor emission increases. In our experiments, the constructs showed that relatively low FRET signals (530 nm) and relatively high concentrations of the monosaccharide had to be applied; however, the ratios between the emission peak highs (480/530) that are dependent on monosaccharide concentration were perfectly aligned on a graph, with a correlation coefficient close to 1 (Figure 2B). The slope coefficient (×10^–5^) was chosen as a determinant of the interaction between a triple glycocodon and a monosaccharide (Figure 2C); in the text below, the numerals in parentheses denote the slope coefficient ×10^–5^. 

At first, we tested ancient DGG, DAA, and DGA glycocodons; the (DGG)_3_D sequence had the highest response to Rib (92), (DAA)_3_D had the highest response to Rib (79), and (DGA)_3_D had the highest response to Gal (99). In the case of Glc, the maximal response was in the (DAA)_3_D construct (51). In other words, each construct responded similarly for the tested monosaccharides, and (DAA)_3_D gave a slightly lower response. However, according to monosaccharide type, Rib gave the highest response in (DGG)_3_D compared to (DAA)_3_D and (DGA)_3_D, Glc gave the highest response in (DAA)_3_D compared to (DGG)_3_D and (DGA)_3_D, Gal gave the highest response in (DGA)_3_D compared to (DGG)_3_D and (DAA)_3_D, and Man gave the highest response in (DGG)_3_D. The results confirmed that, in the postulated prebiotic conditions, DGG is the best “choice” for Rib, DAA is the best for Glc, and DGA is the best for Gal; however, this does not mean that the sequons interact only with these best monosaccharides.

In the second experiment, we used the same linkers, but aspartic acid was substituted with glutamine (Q). The responses presented the same patterns as those in the previous experiment, but the responses to the monosaccharides were slightly higher in the cases of (QGG)_3_Q and (QGA)_3_Q and lower in the case of (QAA)_3_Q (Figure 2C). The (QGG)_3_Q construct provided maximal responses for Rib (112) and Man (96) when tested against all of the tested linkers assessed in this work. 

In the third experiment, we tested some “modern” glycocodons: the amino acid couples from the library of twenty amino acids presently used in biology, which were found to have the best frequencies in the Gal- and Glc-specific proteins [12]. Asparagine (N) was used for the Gal glycocodon, and Q was used for Glc glycocodons; in other words, the NWS sequon was tested for Gal and QMF, QFS, and QFA were tested for Glc. Surprisingly, (QFS)_3_Q was “the best choice” for Gal and (QFA)_3_Q was the best for Glc. In fact, (QFA)_3_Q had the lowest response to the monosaccharides used but had the highest response to Glc (62) amongst all of the tested linkers (Figure 2C), and (QFS)_3_Q had the highest response to the monosaccharides used and had the highest response to Gal (125). 

Lastly, we tested random linkers as a control experiment; the linkers were designed for other purposes and not for studying glycocodons. Figure 2C shows the results for CDLLIRCINC and RWTGRCMSCR. The linkers had equal summary responses, but CDLLIRCINC was a “good choice” for Glc and RWTGRCMSCR gave a better response to Rib.

## 4. Discussion

The quality of information about protein–glycan interactions available in the PDB is still limited; however, high-quality structures for a few sets of monosaccharide forms connected by a small diversity of glycoside linkages are available in sufficient quantity to study protein–carbohydrate interactions [16]. Hydrogen bonds between the carbohydrate hydroxyl groups and polar amino acids in the binding of carbohydrates by proteins were well recognized from the first structures; however, in the last decade, electrostatic and electronic complementarity between carbohydrates and aromatic residues have been shown to play key roles [17]. In monosaccharide binding sites, tryptophan (W) >> tyrosine (Y) > histidine (H) are greatly preferred, asparagine (N) > aspartic acid (D) >> glutamine (Q) are slightly preferred, and aliphatic residues are disfavored [17]. The CH–π stacking contribution to the overall binding energy ranges from −4 kcal/mol to −8 kcal/mol; currently, stacking CH–π interactions are considered driving forces of protein–carbohydrate complexation [18]. However, the calculated energies in the systems without CH–π interactions are in the range from −0.2 to −3.2 kcal/mol; hence, they can also be important for aromatic amino acid and carbohydrate binding processes [19]. In the enzymes used for glycan synthesis/transformations, weaker interactions that enable the release of small carbohydrate fragments after an enzymatic reaction are possibly preferred [19]. To illustrate this, H and Y residues are preferred in GO and only W426 is close to the catalytic area (Figure 1A). On the other hand, not only the frequencies of the residues in catalytic/binding sites are important, but directed evolution studies also usually identify residues far from the active site that have significant impacts on activity and function [20]. Conformational dynamics plus “something else” also play important roles. In the glycocodon theory, the frequency of amino acid couples in whole proteins has been studied in accordance with monosaccharide recognition [12]. In light of the bottom-up model, a study was rooted in “primary” proteins composed of G, A, D, and V amino acids; AA and GV couples were identified as the maximal frequencies for the recognition of Glc, GA and AG were identified as that for Gal, and GD and DG were identified as that for Man (Figure 1B) [12]. For example, in the case of GO, the ancient conserved AGAGQGQAA sequence can be found in front of the catalytic Glc-binding location, doubled GGVVDNAA sequon behind the catalytic Glc-binding location, and H516GV(G) inside of the catalytic Glc-binding location (Figure 1A). QAA and GVD can be considered the glycocodons for Glc, (G)QGQ can be considered the glycocodon for Man, and (AG)AGQ can be considered the glycocodon for Gal. In fact, GO from *Aspergillus niger* is specific for Glc, but the catalytic area also accepts Man (1% relative to Glc) and, at very low levels, Gal (0.08%) [21]. 

It seems that CH–π interactions and aromatic residues such as W, Y, and H are important and that aliphatic residues close/next to stacking amino acids can also be relevant in carbohydrate recognition; for example, in the case of GO, the ancient GVG sequence is precisely behind the key catalytic H516, which theoretically forms the HGV(G) glycocodon/sequon (Figure 1A). In light of this, we studied the DAA, DGA, and DGG sequons in response to Rib, Glc, Gal, and Man (Figure 2). Tripled glycocodons were used as linkers, and constructs such as CFP-(DGG)_3_D-YFP were used to study the change in FRET in the presence of the mentioned monosaccharides. For Glc, to our satisfaction, DAA was confirmed as “the best choice” among the tested primary sequons and, similarly DGG was the best for Rib and DGA was the best for Gal (Figure 2c). GDGD was predicted for Man, and, in actuality, the highest response was found for the DGGDGGDGGD linker. The conditions were the same, and the studied linkers only differed slightly in sequence and 3D form. It seems that the primary “prebiotic” model was well designed. The measurements confirmed the controversial glycocodon theory, which was developed for these modelled primary “prebiotic” conditions, where the first polypeptide–monosaccharide interactions were evolved and conserved. The results (see the responses of the monosaccharides in Figure 2c) also correspond well with the known monosaccharide glycation efficiency: Glc << Gal ≤ Man << Rib. Glc is the least reactive, and Rib is the most reactive; therefore, Glc rather than other stereoisomers has emerged as the universal metabolic fuel and Rib has emerged as the RNA component [22]. As can be seen from the GO structure (Figure 1A), in conserved ancient sequons, aspartic acid is often exchanged for glutamine, histidine, or asparagine. When we changed D for Q in the GG, AA, and GA linkers, QGGQGGQGGQ showed the maximal response for Rib and Man amongst all of the tested linkers (Figure 2c). As mentioned above, hydrogen-bonding Q has a much lower frequency in carbohydrate-binding sites than hydrogen-bonding D [17]. Despite Q being observed much less proximally to carbohydrates in the binding centers, it is functional in the glycocodons, and in our experiments, providing “stronger interactions” for Rib and Man, Glc response did not change. A (G)QGQ glycocodon blocks access to the catalytic center of GO. Perhaps Glc more readily “flows” to the catalytic center of GO than Man, and therefore there is strong preference for Glc. Logically, it seems that the substitution of prebiotic D for N, Q, H, Y, W, R, or K is specific for monosaccharide recognition and binding. 

In the third set of experiments, we tested samples from the predicted “modern” glycocodons/sequons for Glc and Gal. For Glc, MF/FM, FS/SF, and AF/FA showed high frequencies in the Glc-binding proteins [12]; we used Q as the third amino acid and tested the QMF, QFS, and QFA sequons. For Gal, WN/NW and WS/SW were among the best measured frequencies [12]; therefore, we tested the NWS glycocodon. Surprisingly, the QFS glycocodon had the highest response for Gal and QFA had the highest response for Glc amongst all of the tested linkers (Figure 2C). (QFA)_3_Q was remarkable because it provided the lowest “summary response” and the maximal response for Glc amongst all of the linkers. It seems that the substitution of one alanine with phenylalanine in the DAA/QAA ancient Glc glycocodon was convenient in the evolution of protein–Glc interactions; the response increased from 51 for DAA and QAA to 62 for QFA (Figure 2c). The QFS signal increased to 59 in our experiments; in fact, it was previously proposed as a sequon for GlcNAc. The GA/AG and AG/AA frequencies were measured as the maxima for Gal and GlcNAc, respectively [12,13]. This could explain why the QFS glycocodon is among the best glycocodons for Gal recognition in our experiments. QMF surprisingly did not increase but, contrarily, lowered the response to Glc compared to DAA/QAA. It seems that Q is not suitable as a third amino acid for this Glc glycocodon. In the case of (NWS)_3_N, Gal was preferred, but the response decreased compared to DGA/QGA; however, the response to other monosaccharides decreased further. It seems that, in modern glycocodons, the preference is balanced between maximal specificity and maximal “response”.

The random CDLLIRCINC linker, used as a control experiment, showed exactly the same response for Glc as the (QAA)_3_Q linker. The IL/LI amino acid couple has been recognized among the best frequencies for Glc [12]; it seems that the aliphatic LI couple in combination with positively charged arginine (R) could be a regular modern glycocodon for Glc. For example, the LIR95 sequon can be found close to the catalytic center of GO (Figure 1A).

## 5. Conclusions

The stacking CH–π interactions provided by amino acids such as W, Y, or H are essential for protein–monosaccharide complexation in lectins or glyco-enzymes; however, aliphatic residues and other amino acids next to these key aromatic amino acids, currently considered “unfavorable”, are very important for monosaccharide recognition, transport, and binding. Polar hydrogen-bonding residues such as D and N may partly substitute aromatic acid in short-sequence sequons, which recognize and respond to monosaccharide type. The sequons, called glycocodons, are composed of one aromatic (W, Y, or H) or polar (D, N, or Q) amino acid and one amino acid couple convenient for monosaccharide recognition. In prebiotic chemistry, when only the G, A, D, and V amino acids are available, the first glycocodons evolve: DAA(A) and DGV(G) for Glc, DGG(G) for Rib, DGA(G) for Gal, and (D)GDG for Man. Interestingly, these prebiotic sequons/glycocodons are still evolutionarily conserved and can be found in monosaccharide-specific proteins, for example, H516GV(G), Q463AA(A), and AGAGQGQ347AA in GO from *Aspergillus niger* (Figure 1A).

The FRET analysis confirmed that, in prebiotic chemistry, “the best choices” were DAA for Glc, DGG for Rib, DGA for Gal, and DGDG for Man. 

In future, it is necessary to study how these prebiotic glycocodons evolved into modern glycocodons. For example, in the case of QAA(A), the change into QFA markedly improved Glc recognition, as is shown in the presented results.

## Data Availability

Not applicable.
